# Tamoxifen-induced acute eosinophilic pneumonia in a breast cancer patient

**DOI:** 10.1016/j.ijscr.2019.02.026

**Published:** 2019-02-27

**Authors:** Eiyoung Kwon, Mijin Kim, Eunhye Choi, Youngsam Park, Cheolseung Kim

**Affiliations:** Department of Surgery, Presbyterian Medical Center, Jeon-ju, Republic of Korea

**Keywords:** Breast cancer, Tamoxifen, Eosinophilic pneumonia

## Abstract

**Introduction:**

Tamoxifen is often used as antihormonal therapy in patients with breast cancer. However, it has various side effects, of which pneumonia is a rare occurrence.

**Presentation of case:**

A 46-year-old female patient with breast cancer underwent surgical treatment. Tamoxifen was administered as adjuvant therapy on post-operative day 14; 2 days after administration of tamoxifen, the patient developed high fever of more than 39 °C and cough with dyspnea. Based on chest computed tomography findings of ground glass opacity, interlobular septal thickening, and mild pleural effusion in both lungs, eosinophilic pneumonia was suspected. Tamoxifen was discontinued and methylprednisolone injection was administered; the patient showed improvement of symptoms and radiographic findings.

**Discussion:**

Tamoxifen was suspected as the cause of eosinophilic pneumonia since the patient developed high-grade fever at the time of tamoxifen administration, which subsided after discontinuation of the treatment. Other factors considered as the cause of pneumonia were examined, but all showed negative results. In order to confirm tamoxifen as the cause of pneumonia, tamoxifen treatment was restarted at follow-up (post-operative day 47); however, after 1 month, regular administration was not possible due to the development of itching symptom and difficulty in obtaining the patient’s cooperation.

**Conclusion:**

The study highlights that if the patient on tamoxifen develops high fever and cough with dyspnea at 2–3days after the first administration, tamoxifen-induced pneumonia should be suspected.

## Highlights

• If a patient who is taking tamoxifen coughs, be aware of the possibility of pneumonia caused by Tamoxifen.
• Tamoxifen is one of the cause of drug-induced lung injury.

## 1 Introduction

The incidence of breast cancer in Korea is increasing, and the incidence of estrogen receptor (ER) positive breast cancer increased from 58.2% in 2002 to 73.7% in 2015 [1]. Tamoxifen is a selective estrogen receptor modulator that can be used to increase the treatment effect in hormone receptor (HR) positive breast cancer diagnosed prior to menopause and metastatic or recurrent HR positive breast cancer [1]. Various side effects of tamoxifen such as weight gain, sexual dysfunction, hot flashes, neurocognitive deficits, thrombogenic events, and ocular events have been reported [2]. However, tamoxifen-induced pneumonia is a very rare side effect [3].

Herein, we present a case of acute eosinophilic pneumonia after tamoxifen administration in a patient with ER positive breast cancer patient who underwent mastectomy. This work is reported in line with the SCARE criteria [13].

## 2 Presentation of case

A 46-year-old female patient presented to our outpatient clinic for further evaluation of a mass in the left breast that was detected incidentally at a routine health screen in February 2018. She was transferred from the intensive care unit at another center where she was admitted for the treatment of exacerbated epilepsy. Anamnesis revealed a history of mental retardation and medication for epilepsy, but no history of allergy, smoking, and surgery; family history indicated that her sister had received treatment for breast cancer. Ultrasound-guided core needle biopsy performed at our center confirmed a diagnosis of invasive ductal carcinoma. Positron emission tomography–computed tomography and breast ultrasound showed the absence of axillary lymph node involvement, and breast magnetic resonance imaging could not be performed due to the patient’s lack of cooperation. Skin-sparing mastectomy and reconstruction with silicone was performed on April 11, 2018. Histology indicated invasive ductal carcinoma with a mass of 26 × 15 × 15 mm and metastasis in 9 of 10 axillary lymph nodes. The cancer stage was IIIA with T2N2aM0, with ER 8(5 + 3), PR 8(5 + 3), c-erbB2(1+/3), and KI-67(10%).

Treatment with tamoxifen (20 mg/daily) was started on post-operative day 14. On post-operative day 16 (day 3 of tamoxifen treatment), she showed no symptoms other than fever of 39.1 °C. The results of investigation to determine the cause of fever were normal (WBC 6.6 × 10^3^/uL, eosinophil 0.6%) except for mild elevation in the C-reactive protein (CRP) level of 1.13 mg/dL. Culture test for wound infection, using 7 mL of fluid obtained under ultrasound guidance was negative.

Since wound infection could not be completely ruled out as a cause of fever, rifampin (150 mg twice daily) was administered. However, there was persistence of fever of above 39 °C on post-operative day 19 (day 6 of tamoxifen treatment), and absence of redness or pain at the wound site. To rule out antibiotic-related fever, the antibiotic regimen was switched to combined piperacillin and tazobactam, and vancomycin.

**Fig. 1:**
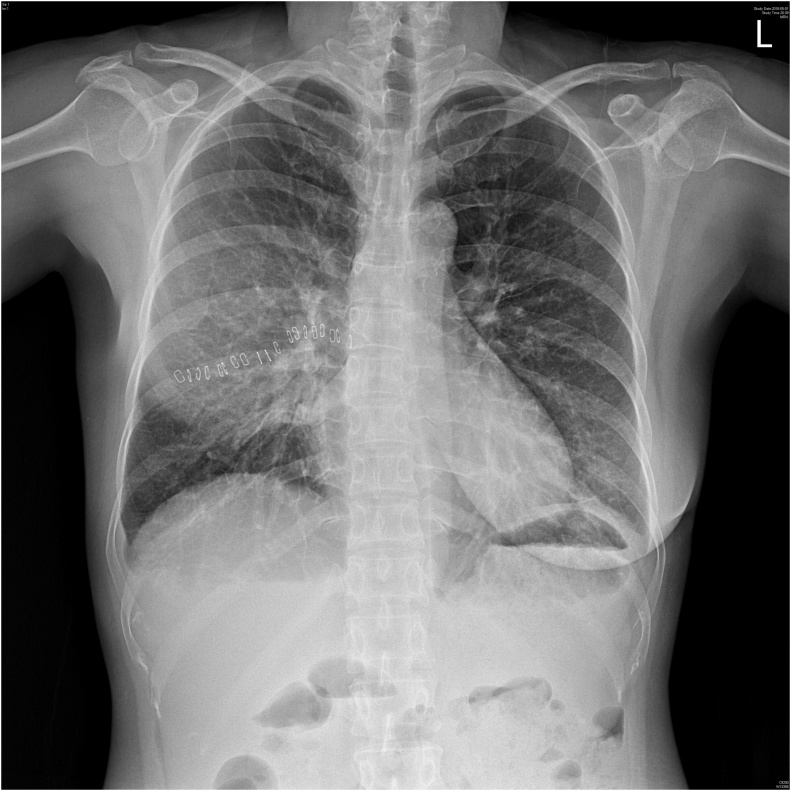
Chest x-ray on post-operative Day 20. Soft haziness in the right middle lung field and costophrenic angle blunting with subsegmental atelectasis in left lower lung field are observed.

On post-operative day 20 (day 7 of tamoxifen treatment), the patient had cough with profuse sputum production and fever of 40.5 °C. Chest radiograph showed soft haziness at the right middle lung field and blunting of the costophrenic angle with subsegmental atelectasis at the left lower lung field [Fig. 1]. Complete blood count showed a marked increase in the level of eosinophils from 0.2% on day 5 to 3.7% on day 7 of tamoxifen treatment, but WBC was not elevated at 4.8 × 10^3^/uL and the CRP level was increased to 7.94 mg/dL.

**Fig. 2:**
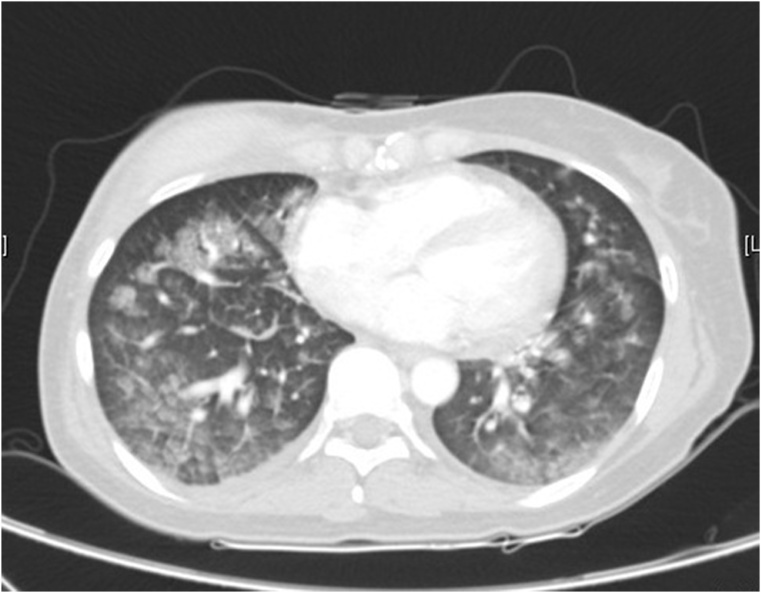
Chest CT on post-operative Day 21. Ground-glass opacification and interlobular septal thickening, and mild pleural effusion in both lungs.

On postoperative day 21(day 8 of tamoxifen treatment), chest computed tomography(CT) was performed, which showed ground glass opacity, interlobular septal thickening, and mild pleural effusion in both lungs. Based on these findings, the patient was diagnosed with suspicious eosinophilic pneumonia [Fig. 2]. She complained of severe dyspnea and showed unstable oxygen saturation of 88% and was transferred to the intensive care unit for close monitoring. The laboratory investigations at the time showed WBC 5.2 × 10^3^/uL, eosinophil 3.6%, and a further increase in CRP level to 8.63 mg/dL. Concomitant blood culture was negative.

Tamoxifen, administered from post-operative day 14, was considered as the probable cause of eosinophilic pneumonia, and discontinued on post-operative day 21 after chest CT was performed. Methylprednisolone (30 mg twice daily) was administered intravenously and tapered by 10 mg every 3 days. Oral methylprednisolone (24 mg daily) was started from post-operative day 29, and reduced dose of the drug (12 mg daily) was administered from post-operative day 33. After the discontinuation of tamoxifen, there was less fever and recovery to normal temperature at day 3 post-discontinuation of tamoxifen. Oxygen saturation was recovered to normal level, and dyspnea was improved. Chest radiographs acquired daily after discontinuation of tamoxifen showed a decrease in haziness, and sputum and cough were reduced.

Mycoplasma pneumonia immunoglobulin M (IgM), *Chlamydia pneumonia* IgM, pneumonia urinary antigen, Gram stain, and polymerase chain reaction for *Mycoplasma tuberculosis* performed in the intensive care unit on post-operative day 22 demonstrated negative results. On postoperative day 33 (day 12 post-discontinuation of tamoxifen), the patient showed improvement of symptoms and was discharged.

## 3 Discussion

Eosinophilic lung disease refers to a condition with an increase in eosinophil count in the peripheral blood or lung tissue [[4], [5], [6]]. Allen and Davis classified eosinophilic pneumonia as an increase in eosinophils in the peripheral blood accompanied by lung infiltration on chest radiography, or eosinophil infiltration through lung histology without an increase in eosinophils in the peripheral blood, or alveolar lavage fluid [4,5]. Eosinophilic lung diseases include simple pulmonary eosinophilia, chronic eosinophilic pneumonia, acute eosinophilic pneumonia, idiopathic hypereosinophilic syndrome, Churg-Strauss syndrome, allergic bronchopulmonary aspergillosis, parasites, and drugs [4,5]. Acute eosinophilic pneumonia can be defined as fever and respiratory distress with myalgia, chest pain, and hypoxia of 1–5 days’ duration that completely resolve without recurrence spontaneously or after the administration of an adrenal cortex hormone in patient without underlying respiratory disease [4,5].

Acute eosinophilic pneumonia shows the following test results: minute interstitial lung infiltration on the chest radiograph that progresses rapidly within 2 days to mixed alveolar and interstitial infiltration, bilateral ground glass opacity and diffuse, reticular densities on CT [6,7]. Acute eosinophilic pneumonia can be caused by drugs. Drugs known to cause acute eosinophilic pneumonia are listed in Table 1 [5,6].

**Table 1:** Drugs causing eosinophilic lung disease.

Ampicillin	Methylphenidate
Beclomethasone dipropionate	Minocycline
Bleomycin	Naproxen
Carbamazepine	Nickel
Chlorpromazine	Nitrofurantoin
Clofibrate	Para-aminosalicylic acid
Cocaine(inhaled)	Penicillin
Cromolyn(inhaled)	Pentamidine(inhaled)
Desipramine	Phenytoin
Diclofenac	Pyrimethamine
Febarbamate	Rapeseed oil
Glafenine	Sulfadimethoxime
GM-CSF	Sulfasalazine
Ibuprofen	Sulindac
Interleukin 2&3	Tamoxifen
Iodinated contrast media	Tetracycline
l-Tryptophan	Tolazamide
Mephenesin carbamate	Tolfenamic acid
Methotrexate	Vaginal sulfonamide cream

Tamoxifen, often used as antihormonal therapy in the treatment of breast cancer, can cause various side effects such as weight gain, sexual dysfunction/loss of libido, hot flashes, neurocognitive deficits, thromboembolic events, ocular events, mood alterations, depression, GI disturbance, bone pain, leg cramps, and insomnia [2]. Pneumonia is a rare side effect of tamoxifen and there are only a few reports of pneumonia in patients who were started on tamoxifen after surgery for breast cancer [[7], [8], [9], [10], [11]].

**Fig. 3:**
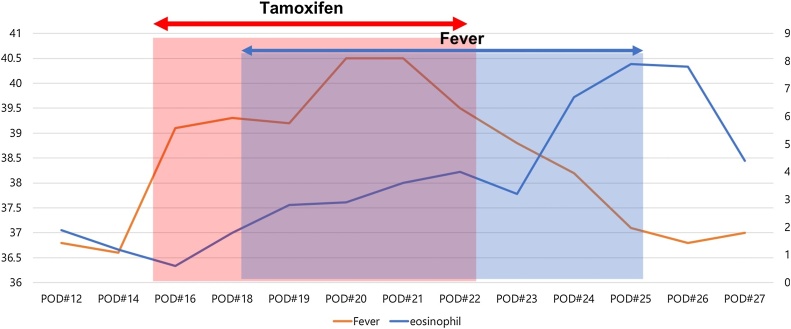
Patient’s body temperature and eosinophil count by tamoxifen administration.

In our case, tamoxifen was considered as the cause of eosinophilic pneumonia due to the association between fever onset and time of first tamoxifen administration. The patient developed high-grade fever of over 39 °C from day 3 of tamoxifen administration, which subsided after the discontinuation of tamoxifen [Fig. 3].

To confirm tamoxifen as the cause of a patient’s pneumonia, tamoxifen should be restarted under patient monitoring for pneumonia recurrence as described in Etori et al. [3]. Previous case reports of tamoxifen-induced pneumonia according to the symptoms, symptom onset-time from medication, and restarting or not are summarized in Table 2.

**Table 2:** Side effects of tamoxifen-induced pneumonia in previous case report.

	Symptom onset at post-medication time point	Symptoms	Restarting	Symptoms after restarting
Ahmed et al. [10]	1week	Cough	No	Not reported
		dyspnea		
		intermittent fever		
		mechanical ventilation		
Shiiki et al. [12]	2day	Cough	Yes	Cough
		dyspnea		dyspnea
Etori et al. [3]	3month	Mild cough	Yes	Cough
				dyspnea on exertion
Kwon et al.^a^	3day	Cough	Yes	Itching sense
		dyspnea		
		Fever (40.5 °C)		

All cases had initial and restarting dose of tamoxifen of 20 mg daily.

a Current study.

In this case, the patient was restarted on tamoxifen 20 mg once daily from May 28, 2018 to confirm tamoxifen as the cause of pneumonia and as choice treatment based on the patient’s age, histology, and pre-menopausal status. Since the patient experienced tamoxifen-related adverse events, alternative therapy with an aromatase inhibitor was considered. Unlike tamoxifen, aromatase inhibitors are only administered after menopause, hence bilateral salpingooophorectomy(BSO) was required in our patient to induce menopause. The option of BSO and aromatase inhibitor was discussed with the patient’s caregiver, and declined based on the concern that the patient’s mental status would prevent her from accepting the consequences of surgery and increase her feelings of loss. Concurrent silicone implantation performed at the time of mastectomy was also due to the caregiver’s concern for the patient’s feeling of loss, though the patient was foreseen to require radiotherapy due to suspicion of axillary lymph node metastasis. However, since the time to natural menopause was too long in our patient, she was administered tamoxifen with methylprednisolone (4 mg twice daily) from May 18, 2018. However, after 1 months’ treatment, regular administration was not possible due to the patients’ symptom of itching and lack of cooperation.

## 4 Conclusion

The current study highlights that in patients undergoing treatment with tamoxifen, if there is development of high-grade fever at 2–3 days after first administration and chest CT shows ground-glass opacification, interlobular septal thickening, and mild pleural effusion, eosinophilic pneumonia due to tamoxifen should be suspected, and tamoxifen should be discontinued. Discontinuation of tamoxifen alone may improve the patient’s symptoms. However, for patients with advanced symptoms, steroid therapy may help to alleviate the symptoms.

## Conflicts of interest

The authors declare that they have no competing interests.

## Funding

This research did not receive any specific grant from funding agencies in the public, commercial, or not-for-profit sectors.

## Ethical approval

In our institute, ethical approval is exempted, depend on acquired patient consent.

## Consent

Written informed consent was obtained from the patient for publication of this case report. A copy of the written consent is available for review by the Editor-in-Chief of this journal.

## Author’s contribution

Kwon EY: writing paper, data collection, table making

Kim MJ : data analysis, figure making

Choi EH : data collection, manuscript review

Park YS : manuscript review

Kim CS : study design, manuscript review

## Registration of research studies

We don’t need to register this work.

## Guarantor

Cheolseung Kim.

## Provenance and peer review

Not commissioned, externally peer-reviewed.
